# Detection of 26 Drugs of Abuse and Metabolites in Quantitative Dried Blood Spots by Liquid Chromatography–Mass Spectrometry

**DOI:** 10.3390/molecules29050975

**Published:** 2024-02-23

**Authors:** Thomas Meikopoulos, Helen Gika, Georgios Theodoridis, Olga Begou

**Affiliations:** 1 Laboratory of Analytical Chemistry, Department of Chemistry, Aristotle University of Thessaloniki, 54124 Thessaloniki, Greece; meikthom@chem.auth.gr (T.M.); gtheodor@chem.auth.gr (G.T.); 2 BIOMIC_Auth, Center for Interdisciplinary Research, and Innovation (CIRI-AUTH), 57001 Thessaloniki, Greece; gkikae@auth.gr; 3 Laboratory of Forensic Medicine & Toxicology, School of Medicine, Aristotle University of Thessaloniki, 54124 Thessaloniki, Greece; 4 ThetaBiomarkers, Center for Interdisciplinary Research, and Innovation (CIRI-AUTH), Balkan Center, 10th Km Thessaloniki-Thermi Rd., P.O. Box 8318, 57001 Thessaloniki, Greece

**Keywords:** LC–MS/MS analysis, drugs of abuse, quantitative dried blood spot, blood micro sampling, drug screening

## Abstract

A method was developed for the determination of 26 drugs of abuse from different classes, including illicit drugs in quantitative dried blood spots (qDBSs), with the aim to provide a convenient method for drug testing by using only 10 μL of capillary blood. A satisfactory limit of quantification (LOQ) of 2.5 ng/mL for 9 of the compounds and 5 ng/mL for 17 of the compounds and a limit of detection (LOD) of 0.75 ng/mL for 9 of the compounds and 1.5 ng/mL for 17 of the compounds were achieved for all analytes. Reversed-phase liquid chromatography was applied on a C18 column coupled to MS, providing selective detections with both +ESI and -ESI modes. Extraction from the qDBS was performed using AcN-MeOH, 1:1 (*v*/*v*), with recovery ranging from 84.6% to 106%, while no significant effect of the hematocrit was observed. The studied drugs of abuse were found to be stable over five days under three different storage conditions (at ambient temperature 21 °C, at −20 °C, and at 35 °C), thus offering a highly attractive approach for drug screening by minimally invasive sampling for individuals that could find application in forensic toxicology analysis.

## 1. Introduction

Dried blood spots (DBSs) represent a minimally invasive sample collection approach that can find applications in various fields. From its introduction in bioanalysis a century ago [1,2,3,4], applications have been increasing over the years, including newborn screening, analysis of small molecules, DNA, proteins for diagnostics [5], therapeutic drug monitoring [6], preclinical drug development, and forensic toxicology [7] among others. The cost-effectiveness, facilitated storage, and shipment conditions of the DBSs in combination with decreased biohazard risk support their potential in the area of toxicological analysis. 

Whole blood, plasma, and urine are the typical specimens used for drug analysis in forensic toxicology [8,9,10,11]. However, shipment, sample storage, and handling of these types of biological samples may bring limitations. DBSs offer numerous benefits, the most important being the ability to collect blood without venipuncture. Reduced invasiveness and low biohazard risk, for instance, for HIV or other infectious pathogens, during sample shipment, are two additional advantages of DBS sampling [12,13]. Moreover, many reports have already focused on the enhanced stability of the analytes in these types of samples at room temperature, without the need for refrigeration [14,15]. 

A crucial challenge, however, is the need for highly sensitive instrumentation and methodologies capable of detecting low levels of drugs in such small blood volumes collected in a DBS. With regard to quantification, the accurate and reproducible collection of blood is often difficult; thus, valid and accurate quantitative data are not feasible. Quantitative dried blood spot (qDBS) analysis overcomes this limitation, offering the advantage of collecting small but precise predefined blood volume, e.g., 10, 20, 50 μL. Thus, it can be used for accurate determination [16,17] besides the screening applications. 

To date, many analytical protocols have been developed to determine/quantify drugs of abuse in DBSs by modern analytical techniques, including liquid chromatography tandem–mass spectrometry (LC–MS/MS) [16,18,19,20,21,22,23,24,25,26] mainly for quantification purposes; liquid chromatography quadrupole time-of-flight mass spectrometry (LC-QTOF-MS) [17,27] and gas chromatography–mass spectrometry (GC–MS) [11,28] for drug screening. Nowadays, various methodologies have been developed for DBSs for both quantification and screening purposes, offering precise analyte level measurements and efficiency of detection in biological samples. Most of these focus on the detection or/and quantification of a specific category of drugs, such as cocaine and its metabolites, benzodiazepines, new psychoactive substances (NPSs), amphetamines, and cannabinoids [14,16,29]. There are few protocols that enable the simultaneous determination of a plethora of illicit drugs belonging to various categories in DBSs but require a minimum blood volume of 15 μL [17,20,21,30,31,32,33]. In all these cases, spots in a simple filter paper, such as Whatman protein saver cards, are used, which presents various limitations, especially in terms of accurate quantification. 

The current study aimed to highlight the opportunities arising from the implementation of qDBSs in drug screening for forensic toxicology purposes. The focus was set on the development of a valuable method for the detection of 26 drugs of abuse and metabolites (both illicit drugs and others) in a 10 μL qDBS that offers high precision in sampling [34,35,36,37,38] and has the potential to be applied for quantitative purposes. The studied substances are those frequently abused. To represent these, we chose the following drugs: benzodiazepines and metabolites (diazepam, bromazepam, temazepam, oxazepam, alprazolam, and 7-aminflunitrazepam), synthetic opioids (methadone and AH-7921), cannabinoids (tetrahydrocannabinol (THC), cannabinol (CBN), cannabidiol (CBD), and synthetic cannabinoids (JWH-018)), cocaine and metabolites (benzoylecgonine, methylecgonine, and cocaethylene), amphetamines analogues (3,4-methylenedioxymethamphetamine (MDMA), 25B-NB2OMe, 25C-NB2Ome, and 25I-NB2OMe), and other stimulants (cathine, mescaline, mephedrone, methylone, 3,4-methylenedioxypyrovalerone (MDPV), and 1-benzylpiperazine). A sensitive qualitative method was developed and evaluated for the detection of the target analytes from qDBSs using liquid chromatography coupled to high tandem mass spectrometry. 

To our knowledge, this is the first method developed for drug abuse screening utilizing a commercially available qDBS device. The proposed protocol delivers efficient and reliable results by using only 10 μL of blood, obtained in a minimally invasive way by finger pricking. 

## 2. Results

### 2.1. LC–MS/MS Optimization

**Figure 1:**
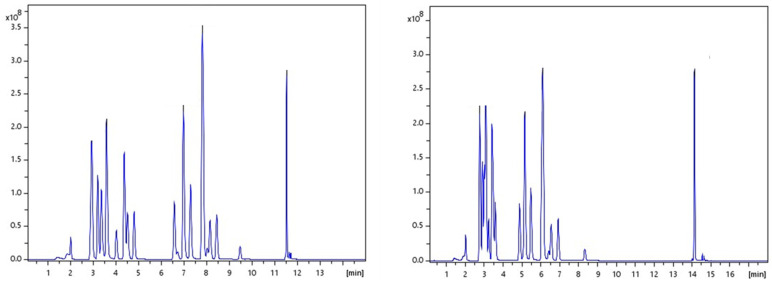
Total ion chromatograms (TICs) of the elution of all 26 illicit drugs in both stationary phases under the same mobile phases and gradient program. The left chromatogram corresponds to Intensity Solo 2 C18 column and the right chromatogram corresponds to Acquity BEH C18.

For the efficient separation of twenty-six (26) drugs and metabolites, some of which have similar structures, various chromatographic systems were tested. Two different C18 columns, an Intensity Solo 2 C18 column (2.1 mm × 100 mm, 2.0 μm) and an Acquity BEH C18 (2.1 mm × 100 mm, 1.7 μm), were used. Mobile phase systems applied included the following: (a) A: H_2_O-MeOH, 90:10 (*v*/*v*), 0.01% FA and B: MeOH, 0.01% FA and (b) A: 5 mM AF in H_2_O-MeOH, 90:10 (*v*/*v*) acidified with 0.01% FA and B: 5 mM AF in MeOH acidified with 0.01% FA. Different gradient elution profiles were tested, starting with up to 95% aqueous phase. Based on the literature, retention of more hydrophilic analytes on RPLC assays requires initial mobile phase conditions of 80–90% water [39]. The optimum selected conditions were finally obtained with A: 5 mM aq. AF-MeOH, 90:10 (*v*/*v*), 0.01% FA and B: 5 mM AF in MeOH, 0.01% FA on an Intensity Solo 2 C18 column. The system demonstrated satisfactory chromatographic performance under all test conditions. However, further improved peak shapes and signals of higher intensity were achieved under these conditions. The chromatographic traces of the analytes obtained on the two columns can be seen in Figure 1. 

Furthermore, to achieve the highest level of sensitivity, detection parameters in the mass spectrometer were optimized. Different MS parameters were tested to achieve the highest intensities and signals for all compounds. Specifically, ion spray voltage for +ESI was set at 5000, while for -ESI at −4500. Cone temperature was set at 300 °C, heated probe temperature at 250 °C, while curtain gas was set at 20 psi. Multiple reaction monitoring (MRM) mode was employed to monitor selective transitions for the target analytes. Two daughter ions for each analyte were selected for detection confirmation. The MRM transitions, retention times, and collision energies for every analyte are listed in Table 1. 

### 2.2. Optimization of qDBS Sample Treatment

In the analysis of DBS samples, the analytes extraction is a crucial step. The applied extraction protocol should be carefully designed to achieve the maximum recovery and sufficient sensitivity levels [40], as this can be challenging due to the small sample volume. During this process, several factors should be considered, including the type of paper used in the device, as there is a possibility of substances being released from its materials during sample extraction [41]. Also, blood hematocrit should be considered, as it has multiple effects on the analyte’s extraction from DBSs. Blood hematocrit determines blood viscosity, which can cause varying DBS homogeneity on the filter paper [42]. In addition, in the field of toxicological analyses and drug screening, several other factors should be taken into account, such as the characteristics of the blood (postmortem or in vivo) and the age of the bloodstains, as they might have a substantial impact on the extraction efficiency [21]. Hence, the validity of the extraction system should be assessed in relation to the aforementioned factors [6,7,12].

Despite the fact that there are numerous methods reported in the literature for the quantitative measuring of drugs in DBS, no data exist yet on the extraction of drugs of abuse from qDBSs. Different paper substrates have been tested, aiming to determine illicit drugs, with Whatman 903 protein saver card being the most commonly used [16,17,18,19,22,23,24,26]. Moreover, Whatman BFC 180 [21], Bond Elute Dried matrix spotting cards (Agilent) [25], FTA DMPK cards [43], and Sartorius Stedim Biotech Sample carrier paper [20] have been examined and proved to be adequately efficient for drug extraction purposes. However, these approaches do not offer the possibility for accurate collection of a specific volume of blood; thus, they have some accuracy limitations in drug analysis.

The Capitainer qDBS device allows for the accurate collection of an exact volume of blood as it is transferred through a capillary to a precut 6 mm paper disc. A more detailed description of the device can be found in a previous work of the authors [38]. 

In the present study, a Capitainer qDBS with two collecting discs (10 μL each) was used. In previously reported studies on the determination of drugs, a variety of samples volume have been used on DBS, in all cases larger than 10 μL. More specifically, either 25 μL [16,17], 30 μL [23,24,43], 50 μL [21,22,26], or 85 μL [18,19] of blood were spotted on paper cards. Hence, the Capitainer qDBS (10 μL of whole blood spotted) is the smallest volume of blood used for such analyses. 

**Figure 2:**
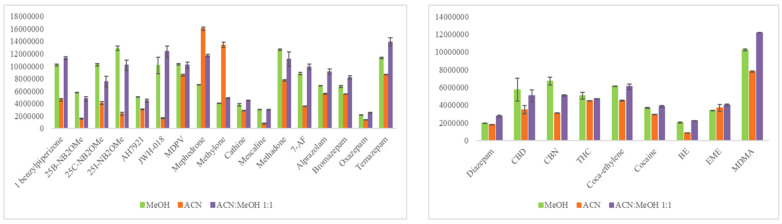
Bar blots illustrating the efficiency of three different extraction solvents for all analytes (x-axes: analyte’s peak area; y-axes: analyte).

The initial step was to design a comprehensive experimental approach to identify the optimum extraction protocol for the analytes of interest from the qDBS sample. Based on our literature findings, several studies in DBS determining either benzodiazepines or cocaine and metabolites, or both, performed SPE [14,18,19]. Extraction solvents tested include MeOH-AcN 3:1 (*v*/*v*) [21,22], pure H_2_O [23], 0.1% FA in MeOH [25], 1% FA in H_2_O [26], MeOH [24], AcN-H_2_O 8:2 (*v*/*v*) [17], and AcN-MeOH 1:1 (*v*/*v*) [43]. Herein, AcN, MeOH, and a mixture of AcN-MeOH, 1:1 (*v*/*v*) were evaluated. An MQC sample (fortified with 50 ng/mL for bromazepam, temazepam, oxazepam, alprazolam, 7-AF, methadone, cathine, mescaline, 25B-NB2OMe, 25C-NB2OMe, 25I-NB2OMe, mephedrone, AH-7921, 1-benzylpiperazine, methylone, MDPV, JWH-018, and with 25 ng/mL for diazepam, cocaine, benzoylecgonine, methylecgonine, cocaethylene, MDMA, THC, CBN, CBD) was used to investigate which would be the most efficient extraction system for all the studied analytes from qDBSs. As can be seen in Figure 2, with the exception of methylone and mephedrone, which were extracted more efficiently with AcN, pure MeOH or mixture of MeOH with AcN provided better extraction recoveries. AcN-MeOH, 1:1 (*v*/*v*) was selected as the optimal qDBS extraction solvent given the fact that 14 out of the 24 drugs had higher intensity using this solvent (in comparison to the other test solvents). Acidification of the AcN-MeOH, 1:1 (*v*/*v*) mixture by adding 0.1% FA, as previously reported [25,26], did not improve recovery and thus was not considered further. One milliliter of solvent provided satisfactory results; higher volumes were tested with no enhanced recoveries.

### 2.3. Drugs Screening

#### 2.3.1. Sensitivity, LOD, and LOQ

The method developed here aims for the application of a minimally invasive sample collection approach for drug screening in blood. Thus, the study was focused on the detection of the drugs of abuse. For this, LOQ and LOD were estimated, as described in Section 3.6.1. Further parameters, such as intra- and interday accuracy and precision related to quantification, were not studied; however, it will be the goal of a future study. For bromazepam, temazepam, oxazepam, alprazolam, 7-AF, methadone, cathine, mescaline, 25B-NB2OMe, 25C-NB2OMe, 25I-NB2OMe, mephedrone, AH-7921, 1-benzylpiperazine, methylone, MDPV, and JWH 018, LOQ was accessed at 5 ng/mL, while for diazepam, cocaine, benzoylecgonine, methylecgonine, cocaethylene, MDMA, THC, CBN, and CBD, at 2.5 ng/mL. Details for LODs are demonstrated in Table 1.

#### 2.3.2. Extraction Recovery (ER%), Hematocrit Effect

Sample volume and hematocrit (Hct) have proved to be two major factors, affecting spot formulation, homogeneity, drying time, and analyte recovery, and are, thus, studied in DBS applications [42,44,45]. In a qDBS device, a precisely measured sample volume is collected on the disc. Nonetheless, the Hct may have an impact on the accuracy of the sampling or, even more so, on the success of the analyte extraction, and, as previously reported [46,47], an independent hematocrit response bias is likely to be observed. 

Herein, the impact of Hct on the extraction recovery of all analytes of interest was investigated by estimating the percentage recovery in three different Hct levels. Results of extraction recovery at LH (35%), FH (40%), and HH (50%) in two different fortified levels are illustrated in Table 2A,B. Based on the results, it was concluded that no effect of Hct was observed, given the fact that ER% spans to similar levels (ranging from 84.6% to 106%) and was within the acceptable criteria [48]. 

#### 2.3.3. Stability

**Figure 3:**
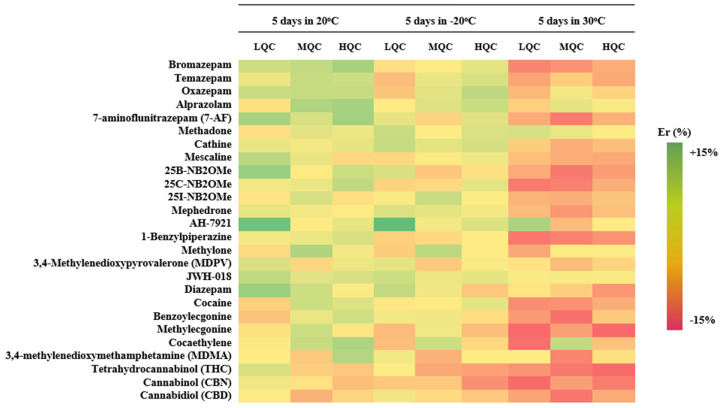
Heatmap illustrating the % Er for all analytes, in three different levels, in three different storage conditions.

Analyte stability was evaluated by analyzing fortified qDBS samples under three different storage conditions (benchtop 20 °C, freezer −20 °C, and oven 30 °C) over 5 sequential days. Results of stability were expressed as % relative error (% E_r_), demonstrated in Table 3. As observed in Figure 3, all analytes were estimated to be within +15% E_r_ to −15% E_r_ in all cases, indicating that the analytes are stable under the given conditions for almost a week allowing for a quite adequate time frame for their analysis. 

## 3. Materials and Methods

### 3.1. Reagents, Materials, and Chemicals

Methanol (MeOH) and acetonitrile (AcN), LC–MS grade, were purchased from HiPerSolv CHROMANORM^®^. LC–MS grade isopropanol (IPA) was obtained from Fisher Scientific International, Inc., Hampton, NH, USA. A Milli-Q purification system (18.2 MΩ cm^−1^) was used to provide ultrapure water. Ammonium formate (AF) ≥99% and formic acid (FA) 98–100% mobile phase additives were purchased from Riedel-de Haën^®^ (Sigma-Aldrich, Steinheim, Germany) and ChemLab, Zedelgem Belgium, respectively. Reference standards of diazepam, bromazepam, temazepam, oxazepam, alprazolam, 7-aminoflunitrazepam (7-AF), methadone, 3,4-methylenedioxymethamphetamine (MDMA), cathine, mescaline, cocaine, benzoylecgonine, methylecgonine, cocaethylene, 25B-NB2OMe, 25C-NB2OMe, 25I-NB2OMe, mephedrone, AH-7921, 1-Benzylpiperazine, methylone, 3,4-Methylenedioxypyrovalerone (MDPV), JWH-018, tetrahydrocannabinol (THC), cannabinol (CBN), and cannabidiol (CBD) were of more than 98% purity and were purchased from Lipomed AG (Arlesheim, Switzerland).

Dried blood spots (qDBSs) devices were obtained from Capitainer AB^®^ (Solna, Sweden).

### 3.2. Working Standard Solutions and Quality Control Samples (QCs)

All analytes’ stock solutions (1 mg/mL) were prepared in methanol by dissolving an appropriate amount of each solid standard. Following dilutions with MeOH, working solutions of 0.1 mg/mL concentration were prepared for each drug. Subsequently, a mixture, containing a concentration of 10 μg/mL bromazepam, temazepam, oxazepam, alprazolam, 7-aminoflunitrazepam (7-AF), methadone, cathine, mescaline, 25B-NB2OMe, 25C-NB2OMe, 25I-NB2OMe, mephedrone, AH-7921, 1-benzylpiperazine, methylone, 3,4-methylenedioxypyrovalerone (MDPV), JWH-018, and at a concentration of 5 μg/mL diazepam, cocaine, benzoylecgonine, methylecgonine, cocaethylene, 3,4-methylenedioxymethamphetamine (MDMA), tetrahydrocannabinol (THC), cannabinol (CBN) and cannabidiol (CBD), was created in H_2_O-MeOH, 50:50 (*v*/*v*). All working and stock solutions were stored at −20 °C.

For validation purposes, a sample prepared by pooling 10 whole blood samples was used (QC). By appropriate spiking of the latter at three different levels of the drugs LQC, MQC, and HQC, samples were prepared, which were then transferred by a syringe onto the qDBS disc. LQC, MQC, and HQC were spiked at 5 ng/mL, 50 ng/mL, and 100 ng/mL, respectively, with bromazepam, temazepam, oxazepam, alprazolam, 7-aminoflunitrazepam (7-AF), methadone, cathine, mescaline, 25B-NB2OMe, 25C-NB2OMe, 25I-NB2OMe, mephedrone, AH-7921, 1-benzylpiperazine, methylone, 3,4-methylenedioxypyrovalerone (MDPV), and JWH-018. For the rest of the drugs, namely, for diazepam, cocaine, benzoylecgonine, methylecgonine, cocaethylene, 3,4-methylenedioxymethamphetamine (MDMA), tetrahydrocannabinol (THC), cannabinol (CBN), cannabidiol (CBD), LQC, MQC, and HQC were prepared by spiking at 2.5 ng/mL, 25 ng/mL, and 50 ng/mL, respectively.

For the evaluation of extraction recovery, venous whole blood collected from three individuals with three different hematocrit levels (low 35%, medium 40%, and high 50%) were used. Spiking at LQC and MQC, as described above for QC, was performed to study the impact of hematocrit in extraction efficiency. The collection of the blood samples was performed under the approval of the Ethical Committee of the Aristotle University of Thessaloniki (protocol number 62883/2023).

### 3.3. Instrumentation and Analytical Conditions

A reversed-phase liquid chromatography–tandem mass spectrometry (RPLC–MS/MS) method was developed for the determination of the 26 drugs in qDBS extracts using an Elute LC chromatographic system coupled to an EVOQ Elite triple quadrupole mass spectrometer (Bruker Daltonics, Bremen, Germany). Separation was carried out on an Intensity Solo 2 C18 (2.1 × 100 mm, 2 μm) column and the mobile phases consisted of A: H_2_O-MeOH, 90:10 (*v*/*v*), 5 mM ammonium formate, 0.01% formic acid and B: MeOH, 5 mM ammonium formate, 0.01% formic acid. Elution was performed by a 15 min gradient as follows: 0–0.5 min: 15–30% B (flow rate 0.2 mL/min), 0.5–10 min: 30–80% B (flow rate 0.2 mL/min); 10–10.5 min: 80–100% B (flow rate 0.4 mL/min); 10.5–12 min 100% B. At 12.01 min, the composition was returned to the initial conditions and column re-equilibration was applied for 3 min. Column temperature was set at 50 °C, and autosampler’s temperature at 4 °C. Injection volume was 5 μL.

### 3.4. qDBS Sample Extraction Optimization

Different extraction conditions were tested to examine the extraction efficiency of the analytes from the qDBS disc. Specifically, extraction recovery and repeatability of various solvents or mixtures, including AcN, MeOH, and AcN: MeOH, 1:1 (*v*/*v*), were assessed. The extraction procedure started by carefully removing one disc (1 × 10 μL) from the Capitainer device, and then by transferring it into an Eppendorf tube. One milliliter of the extraction solvent was added. Vortex-mixing for 10 min, sonication for 10 min, and/or homogenization by bead beater were tested. For the latter, the disc was placed in a tube that contained approximately 20 ceramic bead media balls, vortex-mixed for 10 min, and then was homogenized with solvent for 30 s at a speed of 6.0 m/s; this was repeated twice. In all cases, centrifugation for 10 min at 6700× *g* was thereafter held. Finally, 500 μL of the supernatant were transferred to a tube and evaporated until dryness. The dry residue was reconstituted with 50 μL of H_2_O-MeOH, 85:15 (*v*/*v*). The procedure was performed three times for the different extraction conditions. The solvent system that provided better results was also tested at a smaller volume (200 μL); in this condition, one hundred microliters of supernatant were directly transferred to an LC–MS vial and subjected to analysis.

### 3.5. Final qDBS Sample Treatment Protocol

In a tube that had previously been filled with about 20 ceramic balls (1.4 mm ceramic bead media), one qDBS sample was placed. Then, 1 mL of can-MeOH, 1:1 (*v*/*v*) was added. After 10 min of vortex-mixing, two cycles of beat-beater homogenization lasting 30 s each were carried out at a speed of 6.0 m/s. Five hundred microliters of the supernatant was transferred to a 1.5 mL Eppendorf tube after centrifuged at 6700× *g* for 10 min and evaporated to dryness. The dry residue was reconstituted with 50 mL of H_2_O-MeOH, 85:15 (*v*/*v*).

### 3.6. qDBS Drugs Screening

#### 3.6.1. Sensitivity, LOD, and LOQ

The sensitivity of the method was estimated through the limits of detection (LODs) for the studied analytes. Limit of quantification (LOQ) values were estimated experimentally by analyzing the spiked qDBS HQC sample after serial dilutions. LODs were established as the concentration where the chromatographic peaks-to-noise ratio was 3:1, whereas for LOQ, a 10:1 ratio was considered.

#### 3.6.2. Extraction Recovery (ER%) and Hematocrit Effect

Extraction recovery (ER%) and hematocrit effect were evaluated for the employed extraction protocol. Three blood samples with different hematocrit spanning from low to high levels (low hematocrit, LH 35%; fixed hematocrit, FH 40%; and high hematocrit, HH 50%) were obtained from volunteers to assess the impact of the hematocrit on the extraction efficiency. Two different levels of the analytes standard mixture were added in qDBS samples (LQC, MQC) before and after extraction. Extraction recovery, ER%, was determined based on Equation (1). Hematocrit effect was evaluated as part of extraction recovery efficiency at the three different samples of different hematocrit levels (LH, FH, HH).

(1) $$\%\mathrm{ER}=\frac{\mathrm{Peak}\;\mathrm{area}\;\mathrm{spiked}\;\mathrm{before}\;\mathrm{extraction}}{\mathrm{Peak}\;\mathrm{area}\;\mathrm{spiked}\;\mathrm{after}\;\mathrm{extraction}}\times 100$$

#### 3.6.3. Stability of qDBS Samples

Stability of the analytes in the qDBS samples was studied under three different storage conditions: at benchtop (20 °C), in the oven (30 °C), and in the freezer (−20 °C). Three concentrations (LQC, MQC, and HQC) were examined. Evaluation of short-term stability was carried out by analyzing the spiked qDBS samples (LQC, MQC, HQC) stored under three different conditions for 5 days. The same spiked qDBS samples were analyzed after being freshly prepared to estimate the % relative error (%Er). The three different freshly prepared spiked qDBS QC samples were used to plot a calibration curve, aiding in generating concentration data.

## 4. Conclusions

The applied UHPLC–MS/MS method was developed with the aim to detect 26 illicit drugs in qDBS samples by analyzing only 10 μL of capillary blood. Herein, a simple, rapid, and trustworthy extraction protocol was achieved, reaching high sensitivity levels for all analytes. This is the first approach reported for the detection of frequently screened drugs utilizing a qDBS device, using just a small drop of blood. Stability experiments showed negligible bias that suggests the validity of the method within a 5-day time period, even at RT storage conditions. Therefore, the method offers a great promise for future applications in drug screening for toxicological and forensic analysis purposes.

## Figures and Tables

**Table 1:** MRM transitions, retention times, detection parameters, LOD, and LOQ for all analytes.

Analyte	Molecular Weight	Parent Ion	Daughter Ions	Collision Energy (eV)	Retention Time (min)	LOQ (ng/mL)	LOD (ng/mL)
Bromazepam	316.2	317.0	**228.0**|209.0	40	6.69	5.00	1.50
Temazepam	300.7	301.3	**255.1**|282.9	35	8.42	5.00	1.50
Oxazepam	286.7	287.3	**241.1**|269.1	35	8.02	5.00	1.50
Alprazolam	308.8	309.8	**281.2**|274.1	25	8.13	5.00	1.50
7-aminoflunitrazepam (7-AF)	283.3	284.0	**226.9**|135.1	32	4.48	5.00	1.50
Methadone	309.4	310.0	**265.0**|105.0	14	7.84	5.00	1.50
Cathine	151.2	152.0	**134.0**|117.0	10	2.86	5.00	1.50
Mescaline	211.3	212.0	**195.1**|180.1	22	2.93	5.00	1.50
25B-NB2OMe	380.3	381.0	**121.3**|91.2	25	6.69	5.00	1.50
25C-NB2OMe	335.8	336.8	**121.3**|91.2	20	7.28	5.00	1.50
25I-NB2OMe	427.3	428.3	**121.3**|91.2	20	7.78	5.00	1.50
Mephedrone	177.2	178.1	**160.3**|147.3	10	3.56	5.00	1.50
AH-7921	329.3	329.2	**173.2**|95.3	30	6.56	5.00	1.50
1-Benzylpiperazine	176.3	177.0	**91.2**|85.2	20	3.33	5.00	1.50
Methylone	207.2	208.1	**132.2**|160.3	25	2.91	5.00	1.50
3,4-Methylenedioxypyrovalerone (MDPV)	275.3	276.2	**135.2**|126.3	25	4.35	5.00	1.50
JWH-018	341.5	342.3	**214.4**|155.3	25	11.5	5.00	1.50
Diazepam	284.7	285.5	**154.0**|193.0	28	9.45	2.50	0.75
Cocaine	303.4	304.0	**182.0**|82.0	20	3.99	2.50	0.75
Benzoylecgonine	289.3	290.0	**105.0**|168.0	18	3.58	2.50	0.75
Methylecgonine	199.3	200.0	**82.0**|182.0	18	1.98	2.50	0.75
Cocaethylene	317.4	318.3	**196.2**|82.0	18	4.77	2.50	0.75
3,4-methylenedioxymethamphetamine (MDMA)	193.3	194.0	**163.0**|135.0	12	3.17	2.50	0.75
Tetrahydrocannabinol (THC)	314.4	313.0	**245.2**|191.0	25	11.7	2.50	0.75
Cannabinol (CBN)	310.4	311.2	**223.2**|240.0	20	11.6	2.50	0.75
Cannabidiol (CBD)	314.5	315.0	**193.0**|92.7	25	11.5	2.50	0.75

Daughter ion in bold indicates the quantifier ion.

**Table 2:** (**A**,**B**) Extraction recoveries ± sd in two fortified levels, in three hematocrit levels for the listed analytes.

(A)						
Analyte	Fortified Concentration					
	5 ng/mL			50 ng/mL		
	LH (ER% ± sd)	FH (ER% ± sd)	HH (ER% ± sd)	LH (ER% ± sd)	FH (ER% ± sd)	HH (ER% ± sd)
Bromazepam	85.3 ± 0.5	91.6 ± 0.9	93.9 ± 0.5	89.7 ± 0.8	92.9 ± 1.6	95.2 ± 0.8
Temazepam	90.6 ± 0.4	92.6 ± 0.9	91.7 ± 1.2	90.1 ± 0.6	94.4 ± 1.2	94.2 ± 0.2
Oxazepam	87.7 ± 0.6	89.7 ± 0.2	96.0 ± 0.7	88.4 ± 0.8	93.3 ± 0.3	104 ± 0.6
Alprazolam	90.7 ± 0.4	100 ± 0.5	109 ± 1.7	86.3 ± 0.2	101 ± 1.0	106 ± 1.3
7-aminoflunitrazepam (7-AF)	86.7 ± 0.5	93.7 ± 0.2	87.3 ± 0.7	88.1 ± 1.2	96.1 ± 0.1	96.4 ± 0.5
Methadone	84.6 ± 0.8	105 ± 1.9	99.6 ± 0.7	86.0 ± 0.5	96.2 ± 0.9	94.8 ± 0.3
Cathine	85.7 ± 0.4	89.3 ± 0.4	97.4 ± 0.6	86.2 ± 0.5	88.2 ± 0.6	97.2 ± 0.3
Mescaline	88.4 ± 0.8	94.6 ± 0.6	102 ± 1.0	90.2 ± 0.7	89.8 ± 0.9	98.5 ± 0.7
25B-NB2OMe	89.3 ± 0.7	92.7 ± 0.3	92.9 ± 0.7	88.8 ± 0.6	94.7 ± 0.5	103 ± 1.3
25C-NB2OMe	86.2 ± 0.8	88.7 ± 1.0	94.6 ± 0.8	86.6 ± 0.2	96.6 ± 0.9	88.8 ± 0.8
25I-NB2OMe	86.8 ± 0.1	95.1 ± 0.3	96.9 ± 0.3	89.0 ± 0.4	86.3 ± 0.2	97.4 ± 0.8
Mephedrone	88.9 ± 0.3	89.2 ± 0.6	101 ± 0.8	87.9 ± 0.6	96.4 ± 0.4	95.0 ± 0.1
AH-7921	91.5 ± 0.6	92.7 ± 1.2	88.2 ± 0.4	93.6 ± 0.7	95.5 ± 1.3	99.9 ± 1.1
1-Benzylpiperazine	88.3 ± 1.5	92.6 ± 0.5	85.6 ± 0.3	86.6 ± 0.3	94.8 ± 0.6	93.9 ± 0.4
Methylone	86.5 ± 0.6	88.2 ± 0.4	90.1 ± 0.4	89.0 ± 0.3	92.1 ± 0.4	98.0 ± 0.2
3,4-Methylenedioxypyrovalerone (MDPV)	89.2 ± 0.7	91.5 ± 0.5	96.2 ± 0.3	90.8 ± 0.5	95.0 ± 0.6	97.7 ± 0.8
JWH-018	85.1 ± 0.4	95.8 ± 0.6	88.6 ± 0.4	85.5 ± 0.3	87.3 ± 0.3	90.6 ± 0.4
**(B)**						
**Analyte**	**Fortified Concentration (ng/mL)**					
	**2.5 ng/mL**			**25 ng/mL**		
	**LH** **(ER% ± sd)**	**FH** **(ER% ± sd)**	**HH** **(ER% ± sd)**	**LH** **(ER% ± sd)**	**FH** **(ER% ± sd)**	**HH** **(ER% ± sd)**
Diazepam	92.3 ± 1.2	86.3 ± 0.2	108 ± 1.0	89.1 ± 0.8	90.5 ± 0.8	105 ± 0.8
Cocaine	88.4 ± 0.4	93.4 ± 0.9	102 ± 1.5	85.9 ± 0.5	87.7 ± 0.9	103 ± 1.0
Benzoylecgonine	86.6 ± 0.2	98.7 ± 0.8	94.2 ± 0.2	85.2 ± 0.4	89.5 ± 0.4	91.6 ± 0.8
Methylecgonine	87.9 ± 0.8	105 ± 0.9	94.6 ± 0.8	85.6 ± 0.5	106 ± 0.5	89.7 ± 0.4
Cocaethylene	91.2 ± 0.5	95.6 ± 1.3	91.7 ± 1.2	90.5 ± 0.3	98.4 ± 1.0	88.8 ± 0.8
3,4-methylenedioxymethamphetamine (MDMA)	85.7 ± 0.5	98.7 ± 0.6	96.6 ± 0.9	88.0 ± 0.2	95.6 ± 0.6	94.8 ± 0.3
Tetrahydrocannabinol (THC)	85.7 ± 0.8	87.8 ± 0.6	88.2 ± 0.4	84.8 ± 0.2	96.4 ± 0.2	89.9 ± 0.7
Cannabinol (CBN)	85.2 ± 0.6	89.9 ± 1.1	85.6 ± 0.3	86.3 ± 1.1	88.9 ± 0.5	86.7 ± 1.2
Cannabidiol (CBD)	84.9 ± 0.3	90.4 ± 0.7	87.9 ± 0.4	87.4 ± 0.5	87.3 ± 1.2	89.3 ± 0.6

**Table 3:** Relative error (%E_r_) found for all analytes in three different fortified levels, in three different storage conditions over 5 sequential days.

Analyte	5 Days in 20 °C			5 Days in −20 °C			5 Days in 30 °C		
	LQC (%Er)	MQC (%Er)	HQC (%Er)	LQC (%Er)	MQC (%Er)	HQC (%Er)	LQC (%Er)	MQC (%Er)	HQC (%Er)
Bromazepam	−2.10	−1.20	0.98	−6.77	−5.90	−3.83	−12.9	−12.1	−10.1
Temazepam	−4.40	−1.59	−1.94	−9.17	−4.18	−2.91	−10.8	−7.97	−10.4
Oxazepam	−1.86	−1.60	−1.50	−8.53	−3.80	−0.80	−9.28	−5.00	−7.60
Alprazolam	−6.51	0.20	0.99	−5.94	−3.40	−1.88	−7.85	−4.00	−5.35
7-aminoflunitrazepam (7-AF)	0.80	−2.97	1.20	−4.00	−7.59	−3.62	−10.3	−13.6	−9.92
Methadone	−6.75	−3.78	−4.61	−1.84	−5.78	−3.14	−2.86	−4.38	−5.88
Cathine	−4.33	−5.02	−3.96	−1.57	−3.82	−2.67	−8.01	−10.1	−9.04
Mescaline	−5.81	−4.18	−7.21	−7.25	−5.98	−4.62	−8.92	−10.2	−10.4
25B-NB2OMe	1.88	−5.99	−1.96	−2.97	−8.47	−6.63	−10.3	−13.8	−11.4
25C-NB2OMe	−4.82	−4.38	−0.99	−7.60	−7.17	−3.87	−13.6	−13.2	−10.2
25I-NB2OMe	−6.41	−2.83	−6.67	−5.48	−1.86	−5.73	−9.82	−10.1	−8.53
Mephedrone	−4.12	−5.01	−5.98	−3.16	−4.06	−5.04	−9.22	−11.6	−8.92
AH-7921	5.03	−5.99	−4.16	6.08	−5.05	−3.20	0.63	−9.18	−5.94
1-Benzylpiperazine	−5.04	−4.37	−2.91	−7.80	−7.15	−5.74	−13.8	−13.2	−11.9
Methylone	−7.07	0.20	−4.90	−7.99	−0.79	−5.84	−10.5	−5.96	−6.00
3,4-Methylenedioxypyrovalerone (MDPV)	−2.90	−7.31	−4.55	−3.86	−8.23	−5.50	−6.46	−9.09	−7.43
JWH-018	−1.02	−3.54	−2.94	−2.00	−4.50	−3.90	−5.49	−5.31	−5.49
Diazepam	1.70	−1.98	−5.69	−1.26	−4.83	−8.43	−6.38	−7.91	−11.8
Cocaine	−7.81	−2.00	−3.48	−6.25	−6.00	−3.89	−12.4	−12.1	−10.2
Benzoylecgonine	−8.64	−4.38	−2.21	−4.94	−4.78	−6.84	−11.5	−14.3	−8.25
Methylecgonine	−6.51	−2.02	−6.25	−9.24	−4.87	−8.98	−14.8	−11.1	−14.9
Cocaethylene	−5.49	−1.63	0.40	−9.02	−2.04	−7.24	−14.5	−1.22	−8.85
3,4-methylenedioxymethamphetamine (MDMA)	−5.83	−8.30	−0.20	−4.93	−9.88	−5.79	−5.83	−13.0	−6.59
Tetrahydrocannabinol (THC)	−2.90	−7.95	−8.33	−5.73	−10.63	−11.00	−11.9	−13.8	−14.8
Cannabinol (CBN)	−4.78	−6.64	−8.93	−8.37	−8.30	−12.30	−14.7	−11.2	−13.5
Cannabidiol (CBD)	−6.01	−9.88	−7.37	−4.72	−7.00	−8.37	−10.7	−14.0	−10.4

## Data Availability

Data are contained within the article.
